# Developing a toolkit for patient and public involvement in a clinical trials unit

**DOI:** 10.1186/1745-6215-16-S2-O92

**Published:** 2015-11-16

**Authors:** Heather Bagley, Nicola Harman, Kerry Woolfall, Bridget Young, Hannah Short, Helen Hickey, Paula Williamson

**Affiliations:** 1 University of Liverpool, Liverpool, UK

## 

Patient and public involvement (PPI) in research involves the active contribution of members of the public in research studies and within research organisations. PPI is important in ensuring the relevance and quality of general health care research. Funders often now request evidence of prior and future planned PPI in health research.

The impact of PPI on various aspects of study development and delivery has been described, from study design through to the dissemination and implementation of study findings. Research, however suggests that PPI is inconsistent in terms of levels of engagement, quality and reporting. Within clinical trials variations in intentions and plans for PPI and the experience and impact of such involvement have been identified. Additionally resources to support PPI in clinical trials are often not used in practice. The University of Liverpool Clinical Trials Research Centre PPI working group decided to design a web based toolkit designed to support the PPI activities in clinical trials. The toolkit will signpost chief investigators and trial teams to a range of tools. The tools are designed to facilitate meaningful and effective PPI at all stages of a clinical trial.

The development of the toolkit involves:

• Describing the PPI pathway through a clinical trial

• Identifying existing internal and external resources to support PPI

• Identifying additional required resources

• Evaluating the use the resources

• Disseminating information about the use and impact of the resources and making improvements and modifications to them based on our experience.

The results of developing this toolkit will be presented.

